# Structure and Hierarchy of SARS-CoV-2 Infection Dynamics Models Revealed by Reaction Network Analysis

**DOI:** 10.3390/v13010014

**Published:** 2020-12-23

**Authors:** Stephan Peter, Peter Dittrich, Bashar Ibrahim

**Affiliations:** 1 Department of Fundamental Sciences, Ernst-Abbe University of Applied Sciences Jena, Carl-Zeiss-Promenade 2, 07745 Jena, Germany; stephan.peter@eah-jena.de; 2 Bio Systems Analysis Group, Department of Mathematics and Computer Science, University of Jena, Ernst-Abbe-Platz 2, 07743 Jena, Germany; 3 Department of Mathematics and Natural Sciences, Centre for Applied Mathematics and Bioinformatics, Gulf University for Science and Technology, 32093 Hawally, Kuwait; 4 European Virus Bioinformatics Center, Leutragraben 1, 07743 Jena, Germany

**Keywords:** SARS-CoV-2, Covid-19, corona, within hosts, between hosts, virus dynamics modeling, chemical organization theory, reaction networks analysis, ODEs, PDEs

## Abstract

This work provides a mathematical technique for analyzing and comparing infection dynamics models with respect to their potential long-term behavior, resulting in a hierarchy integrating all models. We apply our technique to coupled ordinary and partial differential equation models of SARS-CoV-2 infection dynamics operating on different scales, that is, within a single organism and between several hosts. The structure of a model is assessed by the theory of chemical organizations, not requiring quantitative kinetic information. We present the Hasse diagrams of organizations for the twelve virus models analyzed within this study. For comparing models, each organization is characterized by the types of species it contains. For this, each species is mapped to one out of four types, representing uninfected, infected, immune system, and bacterial species, respectively. Subsequently, we can integrate these results with those of our former work on Influenza-A virus resulting in a single joint hierarchy of 24 models. It appears that the SARS-CoV-2 models are simpler with respect to their long term behavior and thus display a simpler hierarchy with little dependencies compared to the Influenza-A models. Our results can support further development towards more complex SARS-CoV-2 models targeting the higher levels of the hierarchy.

## 1. Introduction

The current SARS-CoV-2 pandemic has required huge efforts from global society and the scientific community to track, understand, and combat its proliferation. Models of the infection dynamics can help understand SARS-CoV-2 pathogenesis, develop optimal treatments, and introduce appropriate measures to prevent the spread of the virus. There are a multitude of modeling approaches with different properties, applications and aims that can be classed into categories of in-host models (e.g., [1,2,3,4,5,6,7,8]) versus host-to-host models (such as [9,10,11,12]), discrete versus continuous models and ODE versus PDE models (for an overview we refer to [13,14,15,16]). There is an accumulating body of literature on SARS-CoV-2 infection dynamics that make use of these various tools and provide datasets that can be analyzed retrospectively once consensus modeling strategies have been derived [17,18]. The aforementioned models have in common that they rely on an identifiable reaction network, for instance, a set of species and a set of reactions that describe the possible interactions of these species. We have shown that for Influenza A virus infection dynamics [19] reaction network analysis (especially COT [20]) provides metrics to understand, analyze, and categorize different in-host ODE models. In our current work, we applied this to SARS-CoV-2 infection dynamics by extending our previous approach [19] in several directions: We incorporate in-host, host-to-host, and linked models consisting either of ODEs or PDEs. Finally, we combine the models of SARS-CoV-2 with Influenza A in order to compare the dynamics for both viruses. In the next chapter (Materials and Methods) we introduce the herein used method of reaction network analysis by applying it to an example model from Almocera [1]. In the Results chapter, we describe the structural analysis of a set of representative models. For each of these models, we then derive what we call signature. The signature of a model gives a brief overview of its potential dynamical behavior, which allows for relating several models to each other and combining them in a hierarchy. Finally, we link the respective hierarchy of SARS-CoV-2 with that from Influenza A from [19]. In the Conclusions, we summarize our findings and the benefits of the technique applied herein. This novel method can be used as an instrument for a deeper understanding of infection dynamics models and further for an appropriate construction of future virus infection models.

All models, as well as the software tools used to do the analysis, can be found on Github (https://github.com/stephanpeter/orgs-covid).

## 2. Materials and Methods: Procedure for the Organizational Analysis

**Figure 1:**
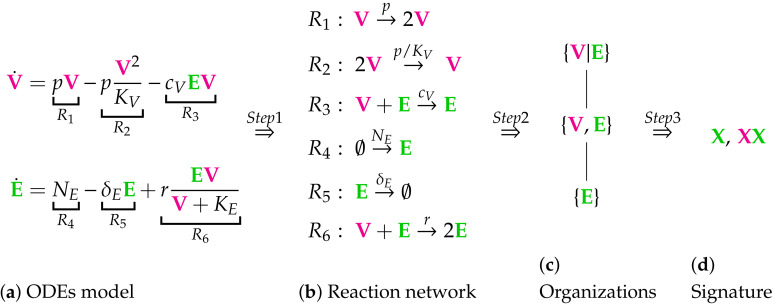
The in-host part of the Almocera Model [1,2] has two variables resp. ODEs (see Subfigure (**a**)): viruses (V) and T-cells (E). This is the starting point of our method consisting of three steps briefly described below. **Step 1**: We derive from the ODE system a set of six reactions (see Subfigure (**b**)): $R_{1},\ldots ,R_{6}$. **Step 2**: We compute from the set of reactions the set of organizations (an organization is a subset of species with specific properties as described below in this Chapter): {V,E} and {E}. We arrange the organizations in a Hasse diagram (see Subfigure (c)), where organizations get bigger from bottom to top and are linked by a line, if the lower organization is a subset of the upper one. **Step 3**: We derive from the set of organizations the signature of the model (see Subfigure (**d**)). For our example, the signature is X,XX, where X represents the organization {E} and XX represents {V,E}. The signature tells us via colored Xs, which of the types (uninfectedorsusceptiblecellsorindividuals, viruses or infected cells or individuals, or immune system, e.g., T-cells) of species are contained in the organizations of the model. We maintain this coloring throughout this work. Note that we use underlining $\underline{X}$ to tag host-to-host species in contrast to in-host species. One should understand the following aspects concerning the method presented above: The long-term behavior of simulations of the dynamics of the model can be easily estimated from the signature: We know from [22] that there is always an organization representing the species persisting in the long-run. Thus species that are not contained in any organization will go extinct for sure after a sufficiently long time period. On the other hand, species that are contained in all organizations of a model, will persist in the long-run for sure. If a species is contained in some organizations of a model but not in all, it has the potential to persist but also to go extinct. It depends on the applied kinetic laws, the initial conditions, and the reaction constants, which case occurs. A hierarchy of a set of models can be constructed relying on their signatures. Like the set of organizations of one model, it can again be visualized as a Hasse diagram. The more combinations of colors a signature contains, the higher is its position in the Hasse diagram and the bigger is the variety of its potential dynamical behavior. If all combinations of colors of one signature are present in a second signature, then the models can be linked by a line. Kinetic laws: Note that except for reaction $R_{6}$, where Michaelis–Menten kinetics are applied, all the other reactions of this example follow mass-action kinetics. The technique of computing and analyzing chemical organizations used in this work applies to both these kinetic laws. Distributed organizations: When the species V and E are separated (we say “distributed”), the reaction $R_{3}$ is inactive. Then, due to the remaining for reactions, the set {V,E} is still self-maintaining and closed and thus some kind of an organization. We write {V|E} (instead of {V,E}) to denote this and call {V|E} a “distributed organization.”

Our method is an extension of that used in [19] to analyze Influenza A virus modeling. We will briefly describe our method for the example of the in-host ODE model from Almocera [1] (see Figure 1).

The method we apply in this work aims at deriving the signature of each model we analyze. Therefore, for each model, the set of organizations must be computed. The computation of the organizations can be divided into two steps:

- Step 1—Deriving the set of reactions: Each summand of each ODE (or PDE) is translated into a reaction as illustrated by the transition from Subfigures (a) to (b) in Figure 1. On the left-hand side of each reaction formula, there is a set of species, the so-called support of a reaction. The support of a reaction is the unique set of species that are needed to run the reaction. If only one of the species of the support of the reaction is missing then that reaction is not active. The term (of the ODE (or PDE)) that belongs to that reaction must be zero if and only if the concentration of at least one of the species in the support of that reaction is zero. The number of the appearance of each species of a reaction on the right-hand side of a reaction is bigger or less than the number on the left-hand side depending on whether the regarding term has a positive or negative sign in the ODE (or PDE)) of the regarding species. As an example we consider reaction $R_{3}$. The corresponding summand is $c_{V}EV$. It is zero if and only if the concentration of at least one of E or V is zero. Thus the support of $R_{3}$ contains exactly the species E and V. On the left-hand side of the reaction equation of $R_{3}$ the species E resp. V appear only to the power of one because of the power of E resp. V is one in $c_{V}EV$. Since the summand $c_{V}EV$ appears only in the ODE of V, namely with a negative sign, the right-hand side of the reaction equation of $R_{3}$ contains one less of V than the left-hand side. The number of E is equal on both sides of the reaction equation since the amount of E is not affected by the reaction $R_{3}$.
- Step 2—Calculating the organizations from the set of reactions: The second step is to compute the organizations (as defined in [21]) from the derived reactions. Each organization consists of a subset of species that is
  1. closed and
  2. self-maintaining.

Before explaining closedness and self-maintenance we have to introduce some other terms regarding reaction networks. Firstly, we discuss reaction equations shortly. As an example we take the reaction

$$\begin{array}{cc} R_{6} & :\;E+V\overset{r}{\to }2V. \end{array}$$

As for every reaction, it contains a reaction arrow, that separates its left-hand side from its right-hand side. The left-hand side, which we call the support of $R_{6}$ (or shortly $supp(R_{6})$), includes one entity of each species E resp. V linked by a plus. The right-hand side of the reaction equation of $R_{6}$ contains two entities of species E.

Now we explain the stoichiometric matrix N of a given reaction network, i.e., of a set of species together with a set of reactions. The stoichiometric matrix $N\in Z^{n\times m}$ consists of *n* lines and *m* columns. Thereby, *n* is the number of species of the reaction network and *m* the number of reactions. e.g., the sixth column of the reaction network of the example Almocera In-host Model can be determined from the reaction equation of $R_{6}$. For each of the two species, the difference of its number of occurrence on the right-hand side minus the number of occurrences on the left-hand side must be calculated. For viruses V we have no appearance on the right-hand side of the reaction equation of $R_{6}$ and one appearance on the left-hand side. Thus the element in the first line and sixth column of *N* equals 0−1=−1. Similarly for E we get for the second line and sixth column of *N* the value 2−1=1.

For a given subset S⫅S of species, a vector $v\in R_{+}^{m}$ of *m* non-negative real numbers $v_{r}$ from

(1) $$\begin{array}{c} R_{+}\equiv \left\{x\in R:\;x\geq 0\right\}, \end{array}$$

one for each reaction r∈R, is called feasible flux if and only if for all reactions r∈R

(2) $$\begin{array}{c} v_{r}>0\Leftrightarrow supp\left(r\right)⫅S. \end{array}$$

We are now able to explain what it means for a subset of species to be closed resp. self-maintaining. These concepts of closedness and self-maintenance stem from chemical organization theory (COT) [23,24].

- A subset S⫅S of species is closed if and only if for each reaction with its support contained in that subset, also all species appearing on the right-hand side of the reaction equation are contained in that subset. In other words, no reaction that is active on the subset *S* produces a species that is not contained in that subset.
- A subset S⫅S of species is self-maintaining if and only if there is a feasible flux for *S* $v\in R_{+}^{m}$ for S such that
  (3) $$\begin{array}{c} N\cdot v\geq 0\in R_{+}^{n}. \end{array}$$

All organizations of a given reaction network can be arranged in a so-called Hasse diagram. For the In-host Almocera example model the Hasse diagram is shown in Figure 1c. From the bottom to the top the organizations have increasing size, indicating an increasing number of species. A line is drawn between two organizations if and only if one is a subset of the other and there is no organization between them. Thus, there is a line between the organizations {E} and {V,E}.

**Figure 2:**
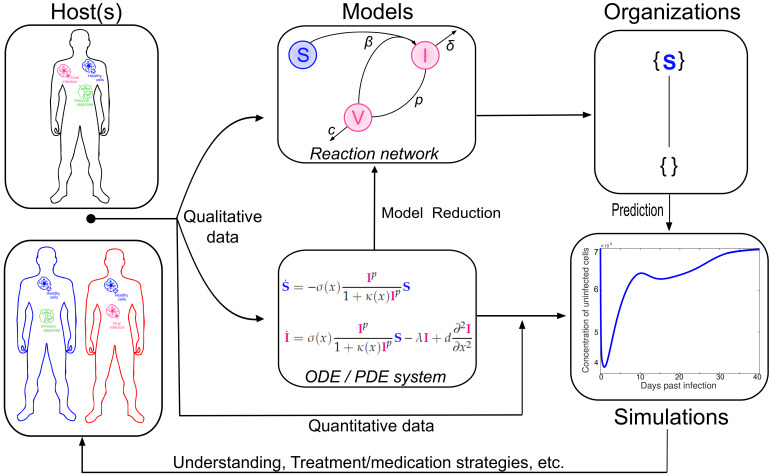
Overview of the relations between organizations and virus dynamics research. The in vivo and in vitro measurements and observations (**left**) lead to quantitative data as well as models (center) concerning the virus dynamics. Those models in turn exist at different scales of abstraction, such as non spatial (like ODEs) or spatial modeling (PDEs) but also in-host (**upper left**) and host-to-host modeling. If a system of partial differential equations can be solved numerically (**right**), which can be challenging, COT provides a tool to further study the dynamics (upper right). When solving the system of partial differential equations becomes too difficult, COT allows for describing the principal components of the system and provides conclusions about its dynamical behavior in simple ways (**right**). Additionally, COT works without any kind of kinetic data, such as reaction or diffusion rates.

In [21] it was proven that for appropriate kinetics, for example, mass-action kinetics, every fixed-point of an ODE is represented by exactly one organization. More precisely, the set of species, that have a strictly positive concentration in the fixed-point, is an organization. Later on, this result was generalized from fixed-points to all kinds of attractors of such dynamical systems [22]. This means that the Hasse diagram of organizations gives an overview of all possible attractors of a dynamical system on the abstract level of the underlying reaction network, regardless of the parameters like reaction constants that were used for the ODEs (or PDEs). Furthermore, COT allows for statements about transitions between those attractors. See Figure 2 for a summary of the fore-mentioned explanations.

Finally, different models with different reaction networks can be analyzed, compared and related to each other in a hierarchy as it was done for Influenza-A virus (IAV) infections [19] and also incorporating the PDEs analysis [25].

In this work, we apply the technique described above to models of SARS-CoV-2 infection dynamics and extend it by including not only dynamical systems

1. consisting of ODEs but also of PDEs,
2. describing in-host dynamics but also host-to-host and mixed (in-host and host-to-host) models.
3. Analyzed infection dynamics of SARS-CoV-2 but also compared to Influenza models

We also show that it is possible to analyze the interrelations between models of different application cases, in this case by ordering Influenza A, SARS-CoV-2, and general virus infection models together in one single hierarchy.

## 3. Analysis of the Models

In this chapter we provide the analysis of all the models listed in Table 1. We provide the respective ODEs and PDEs and the Hasse diagrams of organizations. The reactions that we derive from the models are listed in the Appendix A. We will start with the models basing on ODEs. These models, be they in-host or host-to-host models, were constructed especially for modeling the SARS-CoV-2 infection dynamics. After having analyzed the ODE models, we did the same for the PDE models. Note, that the latter were not solely built to model SARS-CoV-2 infection but rather viral infection dynamics in general. Finally, we analyze one ODE model (the Almocera model [1,2]) linking an in-host scenario (the example model from the Introduction) with a host-to-host scenario of virus infection dynamics.

### 3.1. In-Host Models

**Figure 3:**
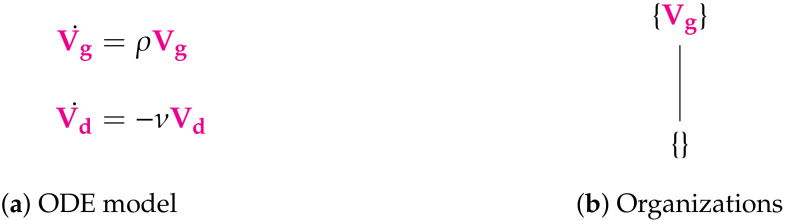
The Vargas-I Model [5,27] with two variables: Exponential growth viruses (V_g_) and decay of viruses (V_d_). There are two organizations: the empty set and the single species set {V_g_}. The set of all species is not an organization since V_d_ decays but is not produced by any reaction. The signature of this model is: ∅,X.

**Figure 4:**
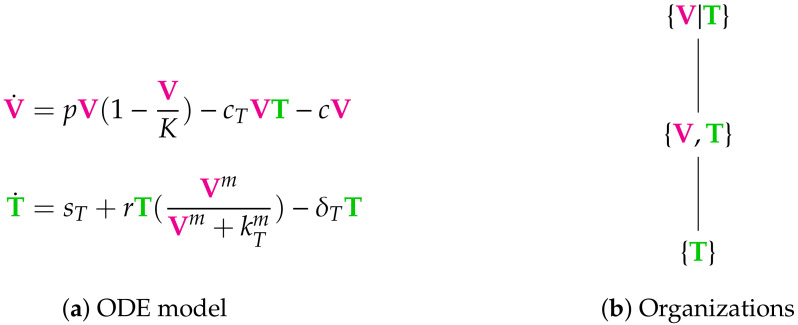
The Vargas-II Model [1,2,5] with two variables: viruses V and T-cells T, and two organizations: {V,T} and {T}. The empty set is not an organization for this model since T has an inflow reaction with reaction constant $s_{T}$ and thus does not go extinct. The organization {V,T} exists also as distributed organization {V|T}. So, if V and T are separated the two reactions with reaction constants $c_{T}$ and *r* are inactive, but this does neither destroy the self-maintenance nor the closedness. The signature of this model is: X,XX. Note that, by replacing T by E in this model, we get almost the same reactions and the same stoichiometric matrix as for the in-host Almocera Model we introduced in the Materials and Methods Section 2.

**Figure 5:**
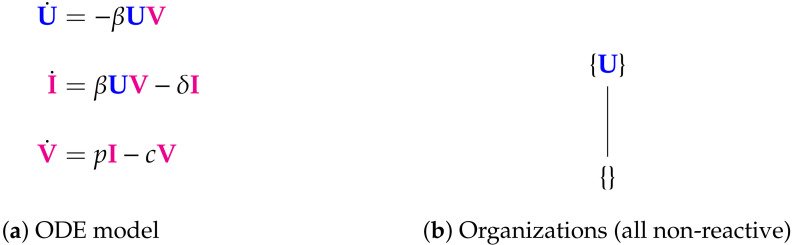
The Abuin Model [5,7,28,29,30] with three variables: susceptible host cells U, infected host cells I, and viral particles V. There are two organizations: the empty set {} and {U}. None of them includes an active reaction; thus we say, that they are “non-reactive”. Note that for this model, a principal part of the infection dynamics, concerning I and V, does not take place within an organization. Thus, from the role organizations play in the long-run of dynamical systems [22,31] we know that this model induces a vanishing of I and V in the long run. The signature of this model is: ∅,X.

**Figure 6:**
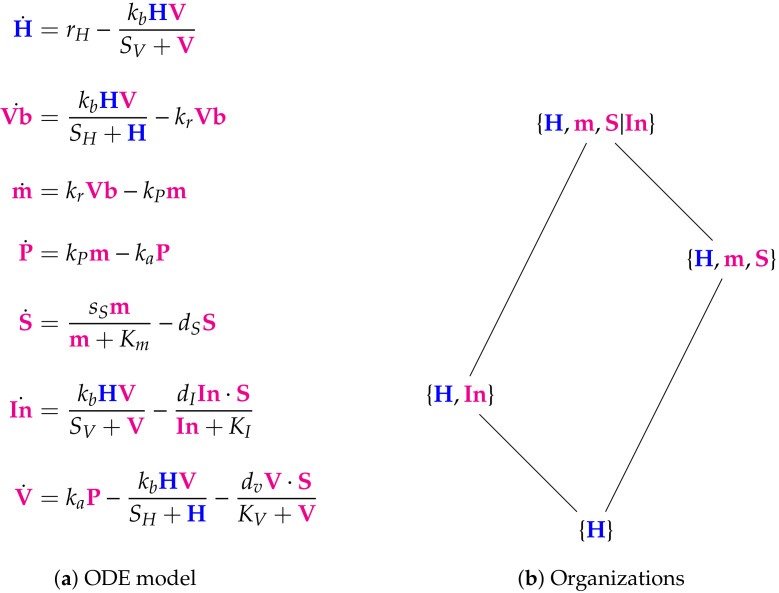
The Su Model [8] with 7 variables: healthy cells H, bound virus Vb, RNA genome m, proteins and replicated RNA packaged together in cytoplasm P, cytokines stimulating inflammatory responses S, infected cells In, coronavirus V. Here we have three organizations and one distributed organization {H,m,S|In} that is not an organization. So, the species H, m, S, and In can only survive together, if S and In are separated. This deactivates the reaction with the reaction constant $d_{I}$ and thus In is able to persist. The signature of this model is: X,XX.

Here we firstly present four in-host ODE models with increasing numbers of species (see Figure 3, Figure 4, Figure 5 and Figure 6). These models describe the spread of the infection within a host, in this case humans. All models contain a virus species but the models differ in terms of the identity of the species.

**Figure 7:**
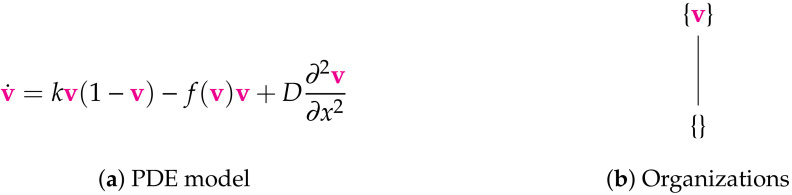
The Bocharov-I Model [6] with one variable: the virus concentration v. There are no boundary conditions specified. Thus, we assume Neumann boundary conditions for simplicity and in the style of the other PDE models analyzed in this work. There is the maximum number of two organizations for a model with one species here. This represents all possible long-term dynamics of the infection, i.e., its persistence as well as its extinction. The signature of this model is: ∅,X.

**Figure 8:**
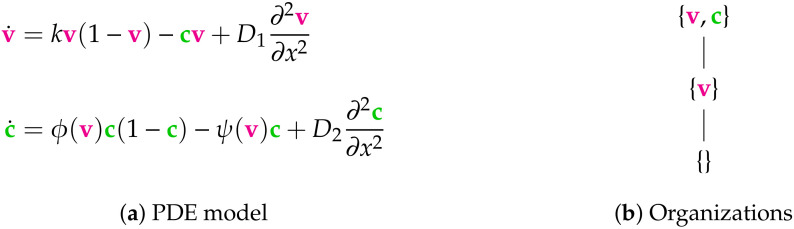
The Bocharov-II Model [5] from the year 2018 with two variables: the virus concentration v and immune cell concentration c. The functions ϕ(v) and ψ(v) are assumed to be strictly positive if and only if v>0. As for the Bocharov-I Model (see Figure 7) we assume Neumann boundary conditions. There are three organizations. Only one subset of species is not an organization, i.e., the set {c}. Thus the model provides a relatively big variety of possible long-term behaviors. The signature of this model is: ∅,X,XX.

Now we analyze two in-host PDE models (see Figure 7 and Figure 8). Contrary to ODE models they are able to deal with spatial inhomogeneities of viral infection processes in the host. They were designed for general viral infections. Thus it is recommended for future SARS-CoV-2 infection dynamics modeling to adapt these models to capture the specifics of this new virus.

Within the set of in-host models we observe a principal difference between the Abuin Model and the other models: The Abuin model does not have an organization with regard to the viral species causing the infection. Thus the Abuin model implicitly assumes the vanishing of the infection over time. The other models do not share this property and thus contain no assumptions regarding viral persistence, which may confer an advantage to these models since it is unclear what the extent of SARS-CoV-2 persistence is. The Su Model exhibits the most complex Hasse diagram. This is the model with the biggest number of species and the only model that explicitly focuses on the genetic aspects of SARS-CoV-2 infection dynamics. Interestingly the Su Model has a distributed organization that is not an organization. This emphasizes the role of the distribution of the species in space or time which is the subject of current research which in turn was recently initiated [25]. Since the Su Model only has ODEs that do not allow for modeling spatial inhomogeneities this is an indication that adapting this model to PDEs may improve the model quality.

### 3.2. Host-To-Host Models

**Figure 9:**
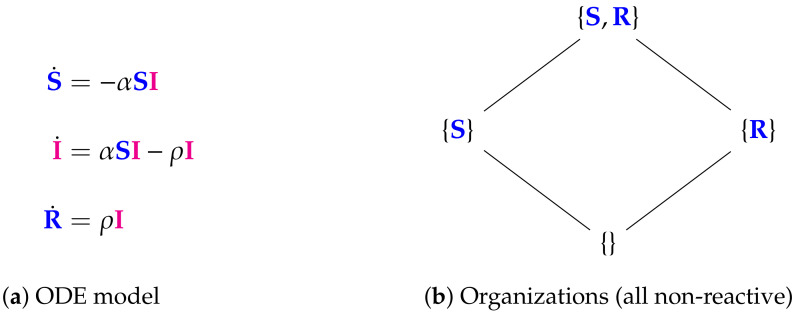
The Nesteruk Model [9,32,33] with 3 variables (SIR): the number of susceptible persons S, infected (sick and infection-spreading) persons I, and removed (sum of isolated, recovered, and dead) persons R. There are four organizations that only contain healthy individuals. None of them contains infected individuals and all are non-reactive, i.e., no reaction is active for these organizations. Thus, as for the in-host Abuin Model from the previous section, the whole infection dynamics takes place outside the organizations. This model inherently assumes that all infected individuals I will vanish finally. The signature of this model is: $\emptyset ,\;\underline{\text{X}}$.

**Figure 10:**
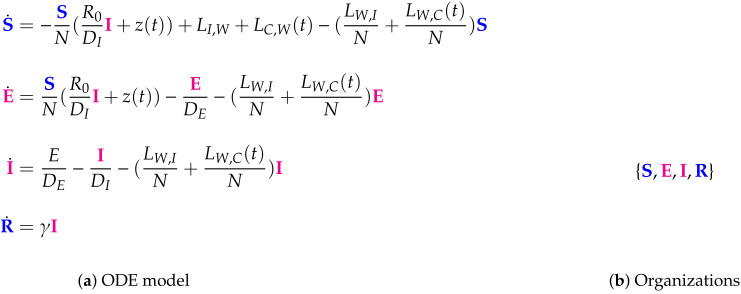
The Wu Model [10] (a SEIR model) with 4 variables: the number of susceptible S, latent E, infectious I, and removed R individuals, and only one organization: {S,E,I.R}, that contains all species. Thus this model implicitly assumes the infection to persist forever once it occurs which is a totally contrary assumption compared to the models assuming the vanishing of infection in the like the previously analyzed Nesteruk model. The signature of this model is: $\underline{\text{X}}\underline{X}$.

**Figure 11:**
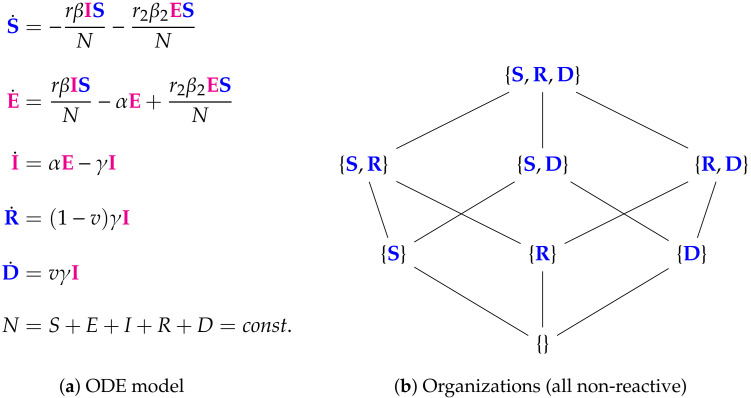
The Bai Model [26,34,35] with 5 variables (SEIRD): the number of susceptible S, exposed E, infected I, recovered R, and dead D individuals. This model has a similar structure in terms of organizations to the Nesteruk model: there is no organization containing species representing the infection. Thus infection is implicitly assumed to vanish in the long-term. The remaining multitude of organizations exists simply due to the fact that recovered individuals R and dead individuals D can be combined arbitrarily with each other and with susceptible individuals S to form organizations. The signature of this model is: $\emptyset ,\;\underline{\text{X}}$.

In this section we first analyze three different host-to-host ODE models describing SARS-CoV-2 infection as it spreads in a human population from one individual to the next (see Figure 9, Figure 10 and Figure 11). Thus these models have three host species in common: susceptible, uninfected individuals (S) and infected individuals (I).

**Figure 12:**
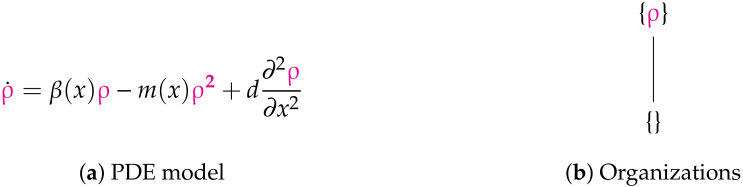
The Fitzgibbon-I Model [11] with one variable ρ representing the current strength of the infection. ρ obeys Neumann boundary conditions. The reaction network structure of this model is almost equal to that of the in-host PDE Bocharov-I model (see Figure 7) from the previous section. Thus there is the maximum number of two organizations. All long-term dynamics of the infection, i.e., its persistence as well as its extinction, are possible. The signature of this model is: $\emptyset ,\;\underline{X}$.

**Figure 13:**
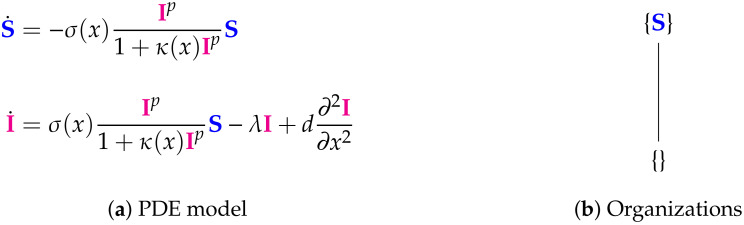
The Fitzgibbon-II Model [11] with two variables: susceptible individuals S(t) and infected individuals I(x,t). Note that only the infected individuals I are modeled dependent not only of time but also of space. It follows Neumann boundary condition. As for the Nesteruk Model and the Bai Model we here have no organization with any species representing the infection. Thus this model implies the infection to go extinct. The signature of this model is: $\emptyset ,\;\underline{\text{X}}$.

The host-to-host PDE models we subsequently analyzed (see Figure 12 and Figure 13) have thus far only been applied to general viral infections. Because of the importance of the spatial dimension in SARS-CoV-2 transmissions, through interventions such as social distancing, it is pertinent to apply this approach to the current outbreak.

The Wu Model and the Fitzgibbon-I Model are the only ones that cover the infection dynamics on the level of organizations. However the Nesteruk Model, the Bai Model, and the Fitgibbon-II Model have solely organizations without species representing infection. Thus these latter (three) models implicitly assume a vanishing of the infection in the long-term. It is currently unclear whether this assumption is justified for SARS-CoV-2.

### 3.3. A Linked In-Host/Host-To-Host Model

**Figure 14:**
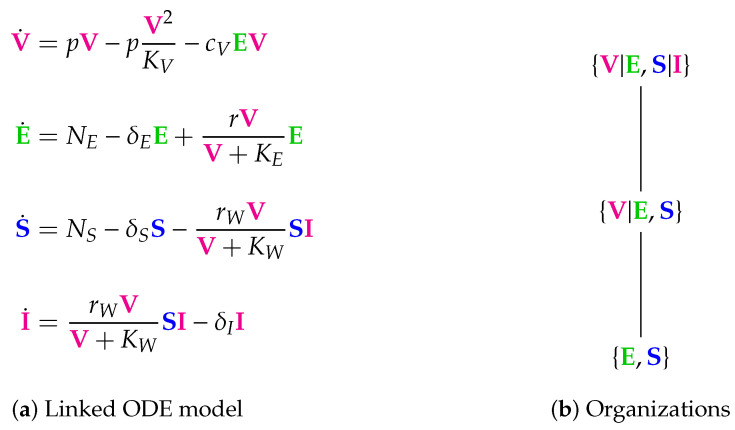
The linked Almocera Model [1,36] has the two variables of the in-host model above (see Figure 1), i.e., viruses (V) and T-cells (E), plus two further variables: susceptible (S) and infected (I) individuals. Note that viruses V as a part of the in-host model influence the host-to-host dynamics via the reaction with reaction constant $r_{W}$. There are three organizations: All of them contain E and S, since they are produced by the two inflow reactions with the reaction constants $N_{E}$ and $N_{S}$, respectively. If V is added to {E,S}, we get the organization {V,E,S} that can exist as a distributed organization by separation of V and E in the same way as it was the case for the in-host model (see Figure 1). If, furthermore, I is added, then we get the full organization where still V and E can be separated, but I must not be separated from V and S since then it could not regenerate vie the reaction with the reaction constant $r_{W}$. Interestingly, in all the organizations of this model in-host species are mixed with host-to-host species. We find that in the long-term the species representing healthy, uninfected hosts, i.e., E and S always persist. Contrarily, V and I might persist too, but might also go extinct. If this is the case for the virus V than also the infected individuals I go extinct. This must not be the case the other way around. Lastly, the COT analysis shows that the model assumes that the T-cells E can exist independently of the virus, but infected individuals can only exist persistently if in contact with healthy individuals S and viruses V. The signature of this model is: $\emptyset ,\;\underline{\text{X}}$. The signature of this model is: $\text{X}\underline{\text{X}},\;X\text{X}\underline{\text{X}},\;X\text{X}\underline{\text{X}X}$.

Here we analyze a model that we called “linked model” (see Figure 14) as it includes in-host as well as host-to-host dynamics, both described by ODEs. This model is designed for viral infections in general and its application to SARS-CoV-2 was deemed of interest because of its bigger focus compared to the previous models. For the analysis of the in-host part of the model we refer the reader to Figure 1 in the Materials and Methods Section 2. In the following we analyzed the linked model, where the in-host model is incorporated into a host-to-host model.

### 3.4. Hierarchy of Models

**Figure 15:**
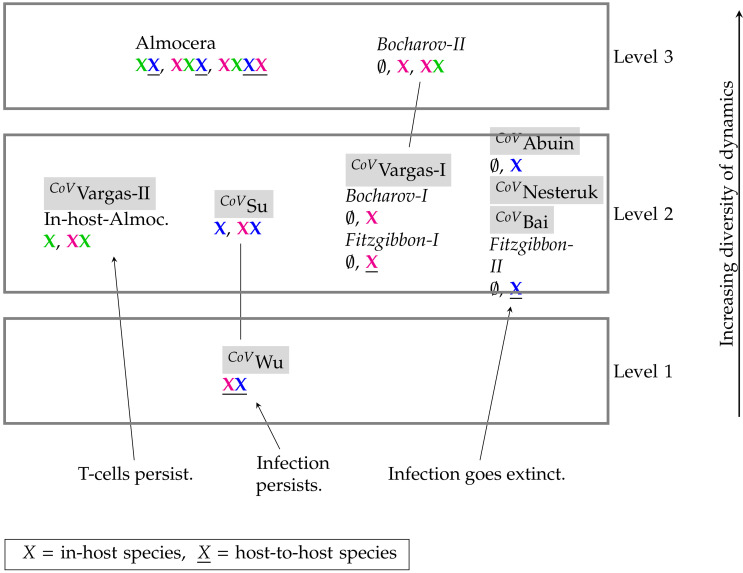
Hierarchy of SARS-CoV-2 models (tagged with *^CoV^*()) and general virus models with respect to their long-term behavior identified by their signature. There are three different levels of increasing signatures. The higher the position of a model in the hierarchy the more diverse is its potential dynamical behavior. By clicking on the model names you are directed to the respective part of this work where the model is analyzed. PDE model names are written in italic.

**Figure 16:**
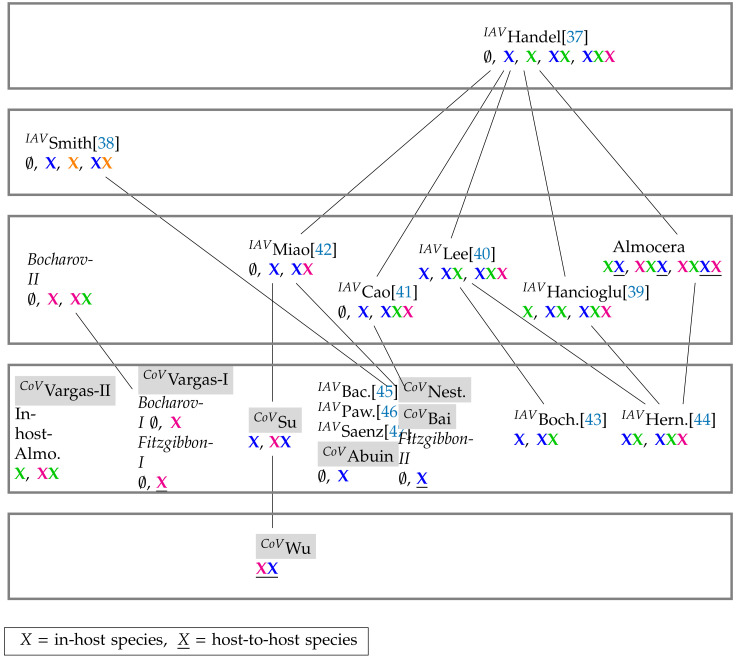
Merged Hasse-diagram of hierarchy of SARS-CoV-2 (tagged with *^CoV^*()), IAV models (tagged with ${}^{IAV}\left(\right)$), and general virus models. They are positioned at five different levels according to the size of their signatures. The higher the level of a model, the bigger is the number of components of its signature and thus the diversity of its potential dynamical behavior. By clicking on the model names you are directed to the respective part of this work where the models are analyzed [37,38,39,40,41,42,43,44,45,46,47]. PDE model names are written in italic. Note, that contrary to many Influenza-A infection models (third level and above) all SARS-CoV-2 infection models are on the second level (counted from bottom to top) and thus have a maximum number of two different species in their signature. This means that they are less complex in terms of their organizations than most of the Influenza-A infection models. Note that the Smith Influenza-A model is the only one that considers co-infection by bacteria X. For more information about the COT analysis of the Influenza-A infection models see [19].

In this section, we present the hierarchies of all models. First, we show the hierarchy of SARS-CoV-2 models (see Figure 15) and then the merged hierarchy of SARS-CoV-2 in addition to IAV models (see Figure 16).

## 4. Conclusions

In this work, we revealed the mathematical structure of different SARS-CoV-2 infection dynamics models operating at different scales of in-host and host-to-host. We have shown how spatial PDE models can be coherently considered as well, resulting in an integrated hierarchical overview of models (Figure 15). This overview can be merged with those of other virus species (here, Influenza-A, Figure 16), which can serve as a beneficial instrument supporting further development of SARS-CoV-2 models.

Additional models similar to those studied here can be easily analyzed and added to the overview, because chemical organization theory can directly be applied to the model’s reaction network structure without requiring further kinetic details. However, complex multi-agent simulation models are problematic, because a corresponding reaction network cannot be derived in a straight forward way.

For each analyzed model we obtained a qualitative description of all possible long-term dynamics. That is, we distinguish sets of species that can persist (organizations) and sets of species that can definitely not persist, no matter what kind of quantitative kinetics are chosen.

We found a number of similarities among the models, for instance, the Abuin, Nesteruk, and Bai model are all in the same group regarding their organizations. Nevertheless, we also found a surprisingly high diversity of models with respect to their long-term qualitative behavior (Figure 15). Interestingly, there is only a small overlap between SARS-CoV-2 and Influenza A models. Furthermore, compared to the Influenza-A models the SARS-CoV-2 models appear to be simpler (mostly level 2) and thus display a simpler hierarchy, i.e., with only one inclusion relation between the Wu and the Su model (Figure 16).

Finally, there is only one model (the Wu Model) implying the unconditional persistence of infected cells. In stark contrast with this, three host-to-host (Nesteruk, Bai, Fitzgibbon-II) and one in-host model (Abuin) imply the definite extinction of any infection in the long-term.

## Figures and Tables

**Table 1:** Overview of all models analyzed in this work each named by its first author and followed by the names of the variables used in the models. By clicking on the model names or the model types (left) you are directed to the part of this work where the respective model is analyzed. The model names tagged with *^CoV^*() in the beginning are explicitly for SARS-CoV-2 infection whereas the others are designed for viral infections in general. All models except for the two models from Bocharov and Almocera (both published in 2018) were published in 2020.

Model Type	ODE	PDE
In-host	[5] *^CoV^*Vargas-I: $V_{g},V_{d}$	[6] Bocharov-I: v
	[5] *^CoV^*Vargas-II: V,T	[6] Bocharov-II: v,c
	[7]*^CoV^*Abuin: U,I,V	
	[8] *^CoV^*Su: H,Vb,m,P,S,In,V	
Host-to-host	[9] *^CoV^*Nesteruk: S,I,R	[11] *^CoV^*Fitzgibbon-I: ρ
	[10] *^CoV^*Wu: S,E,I,R	[11] *^CoV^*Fitzgibbon-II: S,I
	[26] *^CoV^*Bai: S,E,I,R,D	
Linked	[1] Almocera:	
	In-Host: V,E; Linked: V,E,S,I	

**Figure A1:**
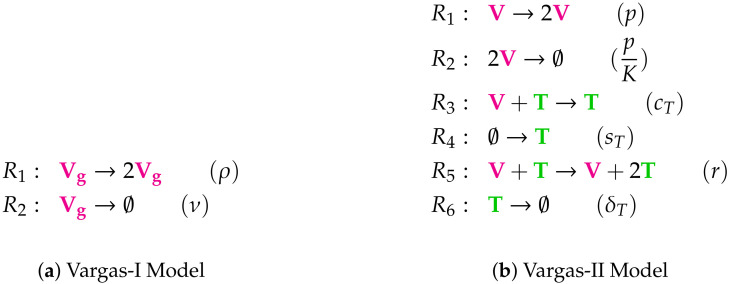
Lists of reactions of the Vargas Models.

**Figure A2:**
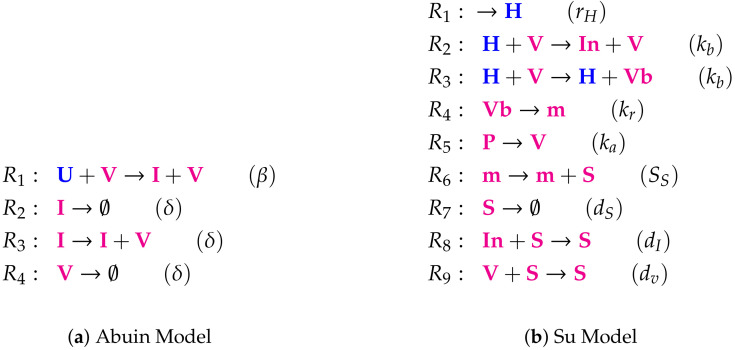
Lists of reactions of the Abuin Model and the Su Model.

**Figure A3:**
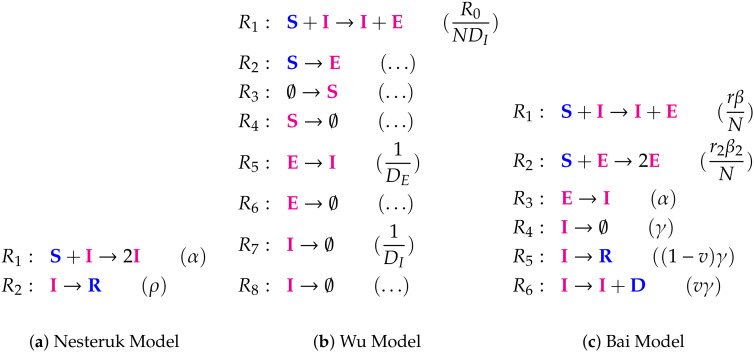
Lists of reactions of the Nesteruk Model, the Wu Model, and the Bai Model.

**Figure A4:**
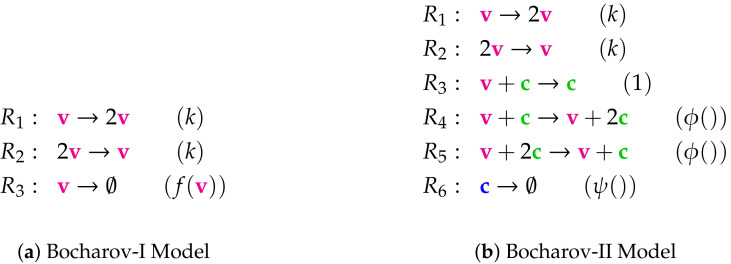
Lists of reactions of the Bocharov Models.

**Figure A5:**
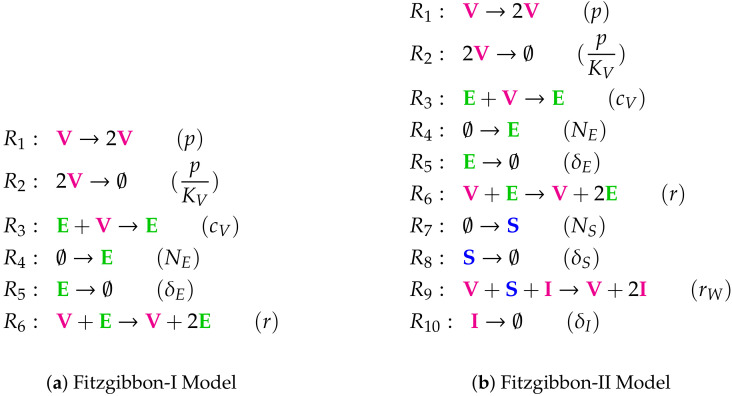
Lists of reactions of the Fitzgibbon Models.

**Figure A6:**
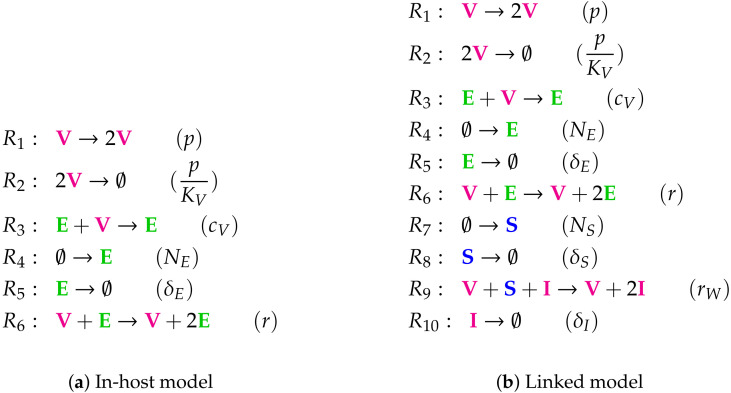
Lists of reactions of the Almocera Model.
